# Eight-year trajectories of changes in health-related quality of life in knee osteoarthritis: Data from the Osteoarthritis Initiative (OAI)

**DOI:** 10.1371/journal.pone.0219902

**Published:** 2019-07-19

**Authors:** Soili Törmälehto, Emma Aarnio, Mika E. Mononen, Jari P. A. Arokoski, Rami K. Korhonen, Janne A. Martikainen

**Affiliations:** 1 Pharmacoeconomics and Outcomes Research Unit, School of Pharmacy, University of Eastern Finland, Kuopio, Finland; 2 Department of Applied Physics, University of Eastern Finland, Kuopio, Finland; 3 Institute of Biomedicine, University of Turku, Turku, Finland; 4 Department of Physical and Rehabilitation Medicine, Helsinki University Hospital, Helsinki, Finland; 5 University of Helsinki, Helsinki, Finland; 6 Diagnostic Imaging Centre, Kuopio, University Hospital, Kuopio, Finland; Monash University, AUSTRALIA

## Abstract

**Background:**

Knee osteoarthritis (OA) worsens health-related quality of life (HRQoL) but the symptom pathway varies from person to person. We aimed to identify groups of people with knee OA or at its increased risk whose HRQoL changed similarly. Our secondary aim was to evaluate if patient-related characteristics, incidence of knee replacement (KR) and prevalence of pain medication use differed between the identified HRQoL trajectory groups.

**Methods:**

Eight-year follow-up data of 3053 persons with mild knee OA or at increased risk were obtained from the public Osteoarthritis Initiative (OAI) database. Group-based trajectory modeling was used to identify patterns of experiencing a decrease of ≥10 points (Minimal Important Change, MIC) in the Quality of Life subscale of the Knee injury and Osteoarthritis Outcome Score compared to baseline. Multinomial logistic regression, Cox regression and generalized estimating equation models were used to study secondary aims.

**Results:**

Four HRQoL trajectory groups were identified. Persons in the ‘no change’ group (62.9%) experienced no worsening in HRQoL. ‘Rapidly’ (9.5%) and ‘slowly’ worsening (17.1%) groups displayed an increasing probability of experiencing the MIC in HRQoL. The fourth group (10.4%) had ‘improving’ HRQoL. Female gender, higher body mass index, smoking, knee pain, and lower income at baseline were associated with belonging to the ‘rapidly worsening’ group. People in ‘rapidly’ (hazard ratio (HR) 6.2, 95% confidence interval (CI) 3.6–10.7) and ‘slowly’ worsening (HR 3.4, 95% CI 2.0–5.9) groups had an increased risk of requiring knee replacement. Pain medication was more rarely used in the ‘no change’ than in the other groups.

**Conclusions:**

HRQoL worsening was associated with several risk factors; surgical and pharmacological interventions were more common in the poorer HRQoL trajectory groups indicating that HRQoL does reflect the need for OA treatment. These findings may have implications for targeting interventions to specific knee OA patient groups.

## Introduction

Osteoarthritis (OA) is the most common type of arthritis and one of the most disabling diseases [1,2]. It is a chronic and progressive disease, which causes joint pain, stiffness, and functional limitations. While the root cause of OA remains unknown, aging, obesity and joint injuries have been found to be major risk factors of OA in the weight bearing joints such as knees and hips [3]. OA in the knees poses a major burden both to individuals and to society. It is estimated that knee OA affects more than 10% of individuals aged over 60 years [4,5]. The societal burden is made up of different costs, for example, joint replacement surgeries, sickness benefits, and disability pensions [6].

Recent research has shown that the knee OA pathway is heterogeneous and patients with knee OA may present with different symptoms [7]. Distinctive subgroups have been identified in the evolution of knee pain and also in the functional limitations experienced by persons with knee OA [8–12]. These studies have modeled trajectories of knee pain intensity, knee pain persistence, and the level and evolution of physical activity limitation. Identifying knee OA symptom pathways and modifiable risk factors related to them could help in the identification of persons who are at risk of suffering a worsening of their quality of life (QoL), and who could benefit from early interventions targeting modifiable risk factors. This would enhance clinical outcomes and allow a more efficient use of limited health care resources. Thus, knowledge about these patient groups and the quantitative impact of each modifiable risk factor in a personalized manner is of major importance.

At an individual level, joint pain, activity limitations, and worsening of QoL are major consequences of knee OA [13–17]. Knee pain, inadequate pain relief and depressive symptoms have been shown to be associated with poor QoL in individuals with knee OA [15–19]. Health-related quality of life (HRQoL) is the health domain of QoL based on subjective experience of one’s health (a patient-reported outcome). Patient-reported HRQoL is considered an important parameter when assessing the outcomes of chronic conditions such as OA [20]. A recent study found three distinct HRQoL groups among persons with knee OA [21]. These groups maintained, however, similar levels of absolute HRQoL scores over time. Very little is known about the trajectories of change in HRQoL related to knee OA. Therefore, our main aim was to model groups of individuals reporting changes in the disease-specific HRQoL measure score compared to baseline to identify those persons with similar HRQoL change patterns related to knee OA by applying a two-stage latent trajectory membership analysis. Trajectory analysis is commonly used to map the evolution of a patient’s symptoms and to investigate differential responses to interventions [22]. Our secondary aim was to evaluate if patient-related characteristics (e.g. age, gender, body mass index, comorbidities), incidence of knee replacements (KR) and the prevalence of pain medication use differed between the members assigned into the different HRQoL trajectory groups. If the identified patient-related characteristics would be modifiable, this could help to plan and target knee OA interventions.

## Materials and methods

### Data sources

Data used in the preparation of this article were obtained from the Osteoarthritis Initiative (OAI) database, which is available for public access at https://data-archive.nimh.nih.gov/oai/. The specific datasets are listed in S1 Table. The OAI is a longitudinal cohort study on knee OA [23]. Subjects in the progression sub-cohort have symptomatic knee OA (frequent knee pain, aching or stiffness with radiographically confirmed knee OA with Kellgren-Lawrence (K-L) grade ≥ 2, in at least one knee) at baseline, while subjects in the incidence sub-cohort have an increased risk of developing knee OA. The specific eligibility risk factors and ethical issues are described in detail in the Osteoarthritis Initiative Study Protocol [23]. Yearly data on HRQoL was used from baseline to the 8-year (96-month) follow-up time point. The incidence of KRs was tracked until the latest update of the database with an extended follow-up time (mean 105 months, standard deviation (SD) 10 months, range 37–119 months).

Data used in the preparation of this article are not proprietary but were obtained from the public OAI database. Ethical approval for collecting this information about the subjects was provided by the OAI, and informed consent was obtained from all individual participants included in the study. This article does not contain any studies with human participants performed by any of the authors. All authors have signed an OAI data use agreement and the study has also been approved by the University of Eastern Finland Committee on research ethics (23/2017).

### Cohort

**Fig 1:**
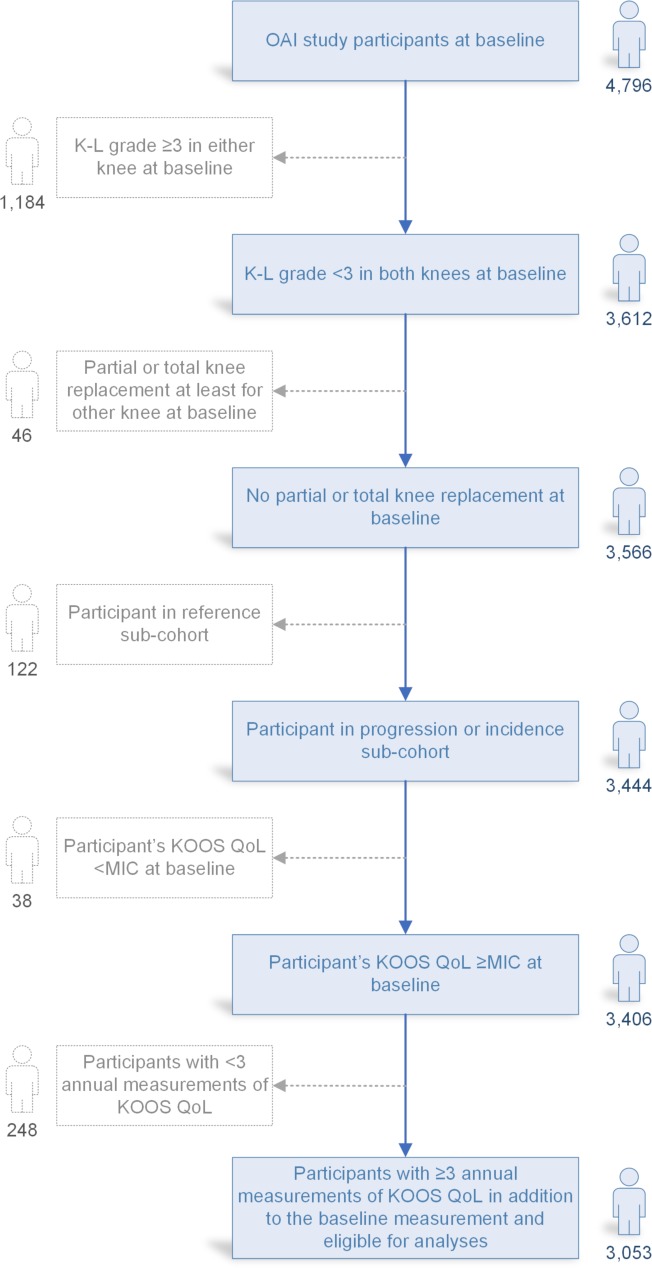
Flow diagram of the participants eligible for the trajectory analyses. K-L grade, Kellgren-Lawrence grade; KOOS QoL, Quality of Life subscale of the Knee injury and Osteoarthritis Outcome Score; MIC, Minimal Important Change; OAI, Osteoarthritis Initiative.

The flow chart of the cohort definition is presented in Fig 1. Briefly, the OAI study participants in the progression and incidence sub-cohorts with no KR at baseline were included. We focused on individuals with mild OA as well as individuals at an increased risk and therefore excluded participants with radiographic K-L grade ≥3. The K-L radiographic system classifies knee OA into five grades: K-L grade 0 indicates an intact joint, K-L grade 2 is considered as the cut-off point of definite OA, and K-L grade 4 indicates severe OA [24]. Individuals having a partial or total KR at least in one knee during the follow-up were excluded after the incident KR as the aim was to track the natural course of early knee OA. Their observations were included in the study data before the KR but after the procedure, they were treated as missing values. Participants with less than three annual follow-up study visits during the 8-year follow-up were excluded in order to decrease the amount of missing data while modeling the trajectories.

### Health-related quality of life

We used the Quality of Life subscale of the Knee injury and Osteoarthritis Outcome Score (KOOS QoL) [25] to quantify HRQoL. KOOS is a measure of knee-associated problems from the patient’s perspective. It is a profile measure incorporating five sub-scales, one being disease-specific QoL, which embodies the person’s experience of HRQoL and daily functioning in relation to both knees together. The KOOS QoL score is based on four questions and a score of 100 indicates no symptoms/problems, while a score of 0 indicates extremely severe symptoms/problems. We used 10 points as Minimal Important Change (MIC) for KOOS QoL [26]. For group-based trajectory modeling, the change in KOOS QoL score in comparison to the baseline score was calculated for each year. The difference was then dichotomized (0 = difference in KOOS QoL >–10; 1 = difference in KOOS QoL scores ≤–10). The group-based trajectory models described later formed the groups based on the dichotomised MIC-variable instead of using the continuous KOOS QoL score.

### Statistical methods

#### Group-based trajectory models

We modeled the annual binary indicators of participants reporting changes in HRQoL (a decrease of ≥10 points, i.e. MIC, in KOOS QoL score) compared to baseline in a logistic group-based trajectory model (GBTM) to identify patients with similar MIC patterns. GBTMs are applied to map the evolution of patient symptoms and to detect if there was a differential response to interventions [22]. GBTMs are an application of finite mixture models using the maximum likelihood method to identify clusters of developmental trajectories [22]. The models produce posterior probabilities of group membership that measure an individual’s likelihood of belonging to the modeled HRQoL trajectory groups given the individual’s changes in HRQoL. Individuals are then placed into the HRQoL trajectory group with the highest posterior probability. There should be a close correspondence between the model estimate of group probabilities and the proportion of individuals classified into the HRQoL trajectory groups. The selection of the final model was based on the Bayesian information criteria. GBTM analyses were conducted with PROC TRAJ [27] in SAS version 9.4 (SAS Institute Inc., Cary, NC). We utilized a two-stage analysis where the response variable (MIC in KOOS QoL) was used to categorize the individuals in the first stage, and in the second stage, the multinomial logistic regression, Cox regression and GEE model were used to identify cross-group differences.

#### Multinomial logistic regression

Multinomial logistic regression was used to investigate the associations between covariates and membership in the defined HRQoL trajectory groups. We used 15 covariates, measured at the baseline visit, as explanatory variables for belonging to the HRQoL trajectory groups. Our main focus of interest was the association between baseline factors and HRQoL trajectory membership and therefore time-varying variables were excluded. Demographics (age, gender, education, living status), clinical status (injuries, surgical history, body mass index (BMI), comorbid conditions, smoking status), and physical activity were selected as a standard set of explanatory variables [28]. We also included race and income into the adjusting variables [29]. Pain and depressive symptoms were selected based on their potential association with HRQoL related to knee OA [15–19]. Finally, the baseline KOOS QoL score was selected as a covariate in order to anchor the effect of baseline HRQoL.

Age and pain were categorized into three groups, and BMI and physical activity were categorized into four and five groups, respectively, to allow for nonlinear associations. Living status, comorbid conditions and depressive symptoms were dichotomized. Education based on the OAI query refers to the highest grade of school completed and had three categories: primary/none (‘less than college’), secondary (‘college graduate’ or ‘some graduate school’), and tertiary level (‘graduate degree’). BMI was based on physical examination.

Knee pain status was based on the OAI question on knee pain severity during the past seven days [12]. For the purpose of the present analysis, the maximum pain score of the participant’s left and right knee was taken into account and categorized into three groups as no pain (score 0–1), mild pain (score 2–3), and moderate pain (score 4–10) [12]. Injury status and surgical history were based on the OAI query on ever injuring either the knee badly enough to limit the ability to walk for at least two days, or ever having knee surgery or arthroscopy.

Physical activity was based on the Physical Activity Scale for the Elderly score, where higher scores indicate greater physical activity [30]. Comorbid conditions were included as the Charlson comorbidity index score [31]. The status of depressive symptoms was based on the Center for Epidemiologic Studies Depression Scale (CES-D) scores dichotomized as no depressive symptoms (score <16) and depressive symptoms present (score ≥16) [9,12].

For this and the remaining statistical analyses, the level of statistical significance was considered as p<0.05. This analysis, along with the following statistical analyses, was conducted with IBM SPSS for Windows, version 23.0.

#### Cox regression

Cox regression analysis was used to estimate the time to KR in relation to membership in the HRQoL trajectory groups to determine if the incidence of KRs differed between these groups. The time between the date of baseline visit and the date of KR (the earliest if ≥1 KR) was measured in months. Participants without a recorded KR event were censored at the last date of a KR recorded in the full cohort. The Cox regression was carried out in an unadjusted mode to test the crude cross-group differences as our aim was to test if the members assigned into the identified trajectories differed with regard to KRs.

#### Generalized estimating equation model (GEE)

We also compared the prevalence of pain medication use between HRQoL trajectory groups during follow-up (from year one forward, baseline visit excluded). The frequent use of pain medication was dichotomized as use or non-use and based on annual OAI queries about using pain medication for joint pain or arthritis on more than half of the past 30 days. As the number of persons using strong pain medication and acetaminophen was low (data not shown), we pooled the answers regarding the use of coxibs, strong prescription pain medications, prescription or non-prescription nonsteroidal anti-inflammatory drugs and acetaminophen due to sample size considerations. A repeated measures generalized estimating equation model (GEE) was used to investigate the associations between medication use and HRQoL trajectory groups [32]. Participants were excluded from the analyses after the incident KR in order to evaluate the same repeated measures in GBTM and GEE models. The model was adjusted for age, gender and baseline medication use to test for cross-group differences. Since our aim was to test if the members assigned into the identified trajectories differed in regard to medication use, we minimized the use of adjusting covariates in order to avoid over-adjusting.

## Results

In total, 3053 OAI study participants were eligible for the present study (Fig 1). The baseline characteristics of OAI study participants are presented in Table 1. A majority i.e. 61% of participants were female and their mean age (SD) was 60.2 (9.0) years. The mean BMI (SD) of the study participants was 28.1 (4.7). The majority of participants did not report any comorbid conditions (78%) or depressive symptoms (91%). Two thirds of the participants reported mild or moderate knee pain. In all, 17.7% of participants belonged to the progression sub-cohort having symptomatic knee OA at baseline. A total of 324 individuals (10.6%) had missing covariate data; these cases were included in GBTM but not in the logistic regression.

**Table 1:** Baseline demographics and clinical characteristics of participants eligible for data analyses.

Variable	Category	N	(%)
**Participants**		3053	
**Cohort**	Incidence	2514	(82.3)
	Progression	539	(17.7)
**Age (years)**	45–54	1008	(33.0)
	55–64	1018	(33.3)
	65–79	1027	(33.6)
	Mean (SD)	60.2	(9.0)
**Gender**	Female	1849	(60.6)
	Male	1204	(39.4)
**BMI**^**a**^	<25	824	(27.0)
	25 to <30	1211	(39.7)
	30 to <35	762	(25.0)
	≥35	254	(8.3)
	Mean (SD)	28.1	(4.7)
	Missing	2	
**Race**	White	2481	(81.3)
	Non-White	569	(18.7)
	Missing	3	
**Education**	None/Primary	1135	(37.4)
	Secondary	936	(30.8)
	Tertiary	965	(31.8)
	Missing	17	
**Income**	<$25k	343	(12.0)
	$25k to <$50k	700	(24.6)
	$50k to <$100k	1067	(37.5)
	$100k or greater	739	(25.9)
	Missing	204	
**Living status**	Living with someone else	2373	(78.2)
	Living alone	660	(21.8)
	Missing	20	
**Knee pain severity**	No pain (0–1)	1001	(32.9)
	Mild pain (2–3)	938	(30.8)
	Moderate pain (4–10)	1108	(36.4)
	Mean (SD)	3.0	(2.6)
	Missing	6	
**Knee injuries**	No	1813	(60.2)
	Yes	1198	(39.8)
	Missing	42	
**Knee surgical history**	No	2602	(85.4)
	Yes	444	(14.6)
	Missing	7	
**KOOS QoL**^**b**^	Mean (SD)	71.0	(20.1)
**Charlson comorbidity index**	0	2337	(77.5)
	≥1	680	(22.5)
	Mean (SD)	0.4	(0.8)
	Missing	36	
**PASE**^**c**^ (quintiles)	0–90	609	(20.1)
	91–134	604	(19.9)
	135–175	608	(20.0)
	176–237	608	(20.0)
	238–526	607	(20.0)
	Mean (SD)	165.1	(82.9)
	Missing	17	
**CES-D**^**d**^	<16	2744	(90.8)
	≥16	277	(9.2)
	Mean (SD)	6.4	(6.8)
	Missing	32	
**Smoking**	Never	1385	(46.0)
	Former	1333	(44.3)
	Current	291	(9.7)
	Missing	44	

Values are *N* (%) unless otherwise noted.

^a^Body Mass Index (kg/m^2^).

^b^Quality of Life subscale of the Knee injury and Osteoarthritis Outcome Score (range 0–100).

^c^Physical Activity Scale for the Elderly (range 0–526).

^d^Center for Epidemiologic Studies Depression Scale (range 0–60).

### Health-related quality of life trajectory groups

**Fig 2:**
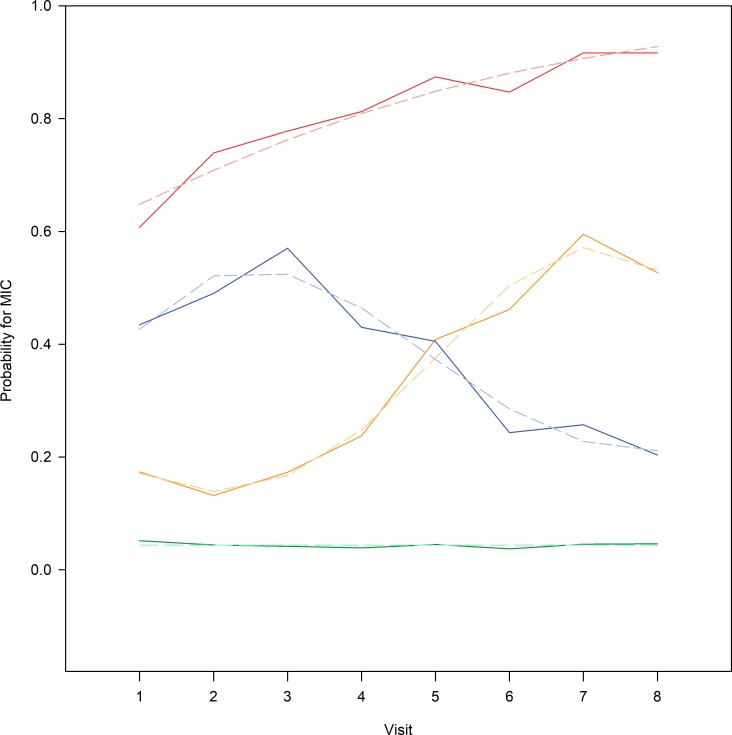
Estimated HRQoL change (MIC) trajectory groups and proportion of individuals in the groups. MIC, a minimally important reduction, i.e. a decrease of at least 10 points, in KOOS QoL. Solid lines represent proportion of individuals classified into the HRQoL trajectory groups and dashed lines represent the model estimate size of the HRQoL groups (lines should be closely corresponding). No change in KOOS QoL, 62.9% (green solid line), predicted 59.5% (lighter green dashed line); improving KOOS QoL after decline, 10.4% (blue solid line), predicted 11.9% (lighter blue dashed line); slowly worsening KOOS QoL, 17.1% (yellow solid line), predicted 19.1% (lighter orange dashed line); rapidly worsening KOOS QoL, 9.5% (red solid line), predicted 9.5% (lighter red dashed line).

Based on Bayesian information criteria values, we selected a 4-group model as the final trajectory model (S2 Table). According to diagnostic criteria, the selected model performed adequately (S3 Table). The estimated four trajectories and the averaged group data are presented in Fig 2. The following HRQoL change patterns were identified: (1) ‘no change’ (estimated size 59.5% of the whole cohort) with virtually no worsening in KOOS QoL, (2) ‘slowly worsening’ (19.1%) where the probability of experiencing a minimally important reduction in KOOS QoL steadily increased, (3) ‘improving’ KOOS QoL after an initial decline (11.9%), and (4) ‘rapidly worsening’ (9.5%) where the probability of experiencing a minimally important reduction in KOOS QoL increased rapidly. The proportion of individuals classified in the ‘no change’, ‘slowly worsening’, ‘improving’ and ‘rapidly worsening’ KOOS QoL groups were 62.9%, 17.1%, 10.4% and 9.5%, respectively, which were in close correspondence with the model estimate of group probabilities mentioned above.

### Association of covariates with HRQoL trajectory groups

Table 2 presents the statistically significant associations of baseline covariates with the HRQoL trajectory groups. We used the ‘no change’ group as the reference in the multinomial logistic regression. Baseline characteristics of participants stratified by trajectory groups are presented in S4 Table.

**Table 2:** Odds ratios (OR) and 95% confidence intervals (CI) of multinomial logistic regression analysis (trajectory of ‘no change in KOOS QoL’ as reference).

		OR (95% CI)		
Variable	Category	Improving KOOS QoL after decline	Slowly worsening KOOS QoL	Rapidly worsening KOOS QoL
**Age (years)**	45–54	1.0	1.0	1.0
	55–64	0.93 (0.67–1.29)	0.98 (0.75–1.28)	1.17 (0.83–1.65)
	65–79	0.94 (0.66–1.36)	1.07 (0.80–1.45)	0.65 (0.44–0.97)
**Gender**	Male	1.0	1.0	1.0
	Female	1.36 (1.02–1.83)	1.11 (0.87–1.41)	1.44 (1.05–1.98)
**BMI^a^**	<25	1.0	1.0	1.0
	25 to <30	1.51 (1.07–2.14)	1.10 (0.84–1.44)	1.92 (1.31–2.81)
	30 to <35	2.02 (1.38–2.96)	1.59 (1.18–2.14)	3.23 (2.15–4.85)
	≥35	2.31 (1.38–3.87)	1.53 (0.98–2.39)	2.04 (1.11–3.77)
**Race**	White	1.0	1.0	1.0
	Non-White	0.93 (0.64–1.33)	0.91 (0.67–1.24)	0.74 (0.50–1.10)
**Socioeconomic status**				
**Education**	Tertiary	1.0	1.0	1.0
	Secondary	0.97 (0.70–1.35)	1.05 (0.80–1.37)	1.01 (0.70–1.45)
	None/Primary	0.99 (0.69–1.40)	1.18 (0.88–1.57)	1.20 (0.83–1.73)
**Income**	$100k or greater	1.0	1.0	1.0
	$50k to <$100k	1.04 (0.74–1.46)	1.02 (0.77–1.35)	1.57 (1.06–2.33)
	$25k to <$50k	0.79 (0.52–1.22)	1.04 (0.74–1.47)	1.84 (1.16–2.92)
	<$25k	1.27 (0.75–2.16)	1.20 (0.77–1.87)	3.03 (1.73–5.33)
**Living status**	Living with someone else	1.0	1.0	1.0
	Living alone	1.04 (0.74–1.45)	1.02 (0.78–1.35)	0.82 (0.57–1.16)
**Clinical characteristics of knee**				
**Knee pain severity**	No pain (0–1)	1.0	1.0	1.0
	Mild pain (2–3)	1.17 (0.82–1.67)	1.01 (0.76–1.34)	1.50 (1.04–2.17)
	Moderate pain (4–10)	1.72 (1.15–2.57)	1.17 (0.84–1.62)	1.96 (1.27–3.03)
**Knee injuries**	No	1.0	1.0	1.0
	Yes	1.03 (0.77–1.37)	1.02 (0.80–1.28)	1.08 (0.80–1.47)
**Knee surgical history**	No	1.0	1.0	1.0
	Yes	1.63 (1.12–2.38)	1.44 (1.05–1.97)	1.29 (0.83–2.01)
**KOOS QoL^b^**		1.04 (1.03–1.05)	1.03 (1.02–1.04)	1.06 (1.05–1.07)
**Other factors**				
**Charlson comorbidity index**	0	1.0	1.0	1.0
	≥1	1.43 (1.05–1.94)	1.07 (0.82–1.40)	1.26 (0.90–1.77)
**PASE^c^**	238–526	1.0	1.0	1.0
	176–237	1.09 (0.71–1.68)	1.04 (0.74–1.45)	1.44 (0.92–2.26)
	135–175	1.19 (0.77–1.83)	1.19 (0.86–1.67)	1.29 (0.81–2.06)
	91–134	1.23 (0.80–1.89)	1.05 (0.75–1.48)	1.12 (0.69–1.81)
	0–90	1.11 (0.72–1.72)	0.61 (0.42–0.89)	1.33 (0.83–2.14)
**CES-D^d^**	<16	1.0	1.0	1.0
	≥16	1.73 (1.11–2.69)	1.54 (1.05–2.24)	1.33 (0.80–2.22)
**Smoking**	Never	1.0	1.0	1.0
	Former	1.13 (0.86–1.49)	1.10 (0.87–1.38)	1.15 (0.85–1.55)
	Current	1.02 (0.62–1.68)	1.34 (0.92–1.97)	2.08 (1.32–3.28)

^a^Body Mass Index (kg/m^2^).

^b^Quality of Life subscale of the Knee injury and Osteoarthritis Outcome Score.

^c^Physical Activity Scale for the Elderly.

^d^Center for Epidemiologic Studies Depression Scale.

Women had higher odds of belonging to the ‘rapidly worsening’ and ‘improving’ KOOS QoL trajectories. Higher BMI at baseline was associated especially with belonging to the ‘rapidly worsening’ and ‘improving’ trajectories. Other clinical factors associated with belonging to the ‘rapidly worsening’ trajectory were smoking and mild or moderate knee pain. In addition, a lower income was associated with belonging to the ‘rapidly worsening’ trajectory. The oldest age group (65–79 years) had lower odds of belonging to the ‘rapidly worsening’ trajectory. Depressive symptoms were associated with higher odds of belonging to the ‘improving’ and ‘slowly worsening’ trajectories. Furthermore, the surgical history was associated with belonging to the ‘improving’ and ‘slowly worsening’ trajectories.

### Association of KRs with HRQoL trajectory groups

**Fig 3:**
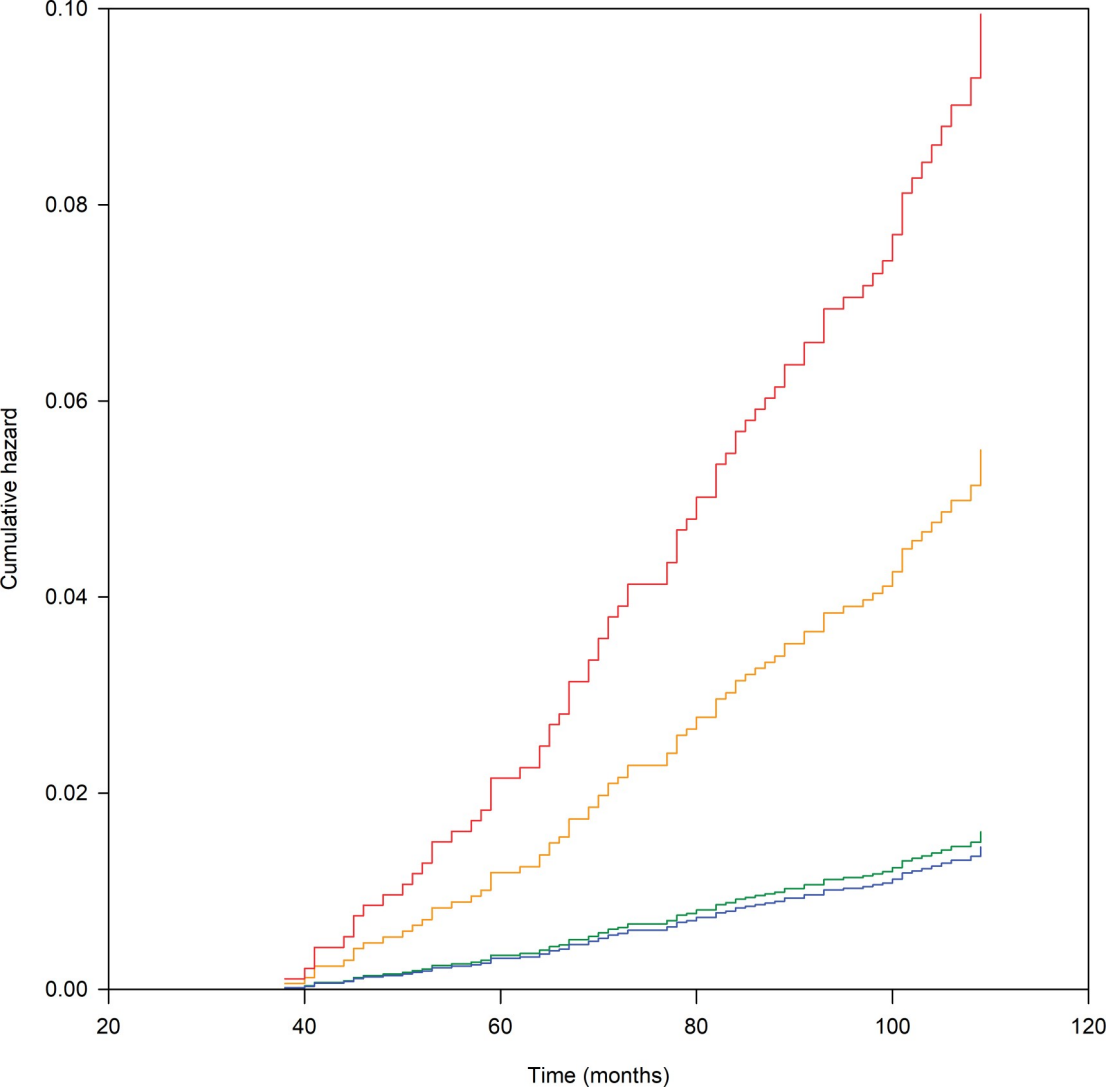
Cox cumulative hazard function for knee replacement during extended follow-up by HRQoL trajectory groups. The time to KR (months between the dates of baseline visit and the date of KR) in relation to membership in the trajectory groups. Participants without a recorded KR event were censored. The Cox regression was carried out in an unadjusted mode. HRQoL change (MIC, a minimally important reduction in KOOS QoL) trajectory groups: no change in KOOS QoL (green line); improving KOOS QoL after decline (blue line); slowly worsening KOOS QoL (yellow line); rapidly worsening KOOS QoL (red line).

The hazard function for KR is presented in Fig 3. The proportion of participants having KR in the ‘no change’, ‘improving’, ‘slowly’ and ‘rapidly’ worsening trajectories was 1.4%, 1.3%, 4.8% and 8.2%, respectively. The ‘rapidly worsening’ (hazard ratio (HR) 6.1, 95% CI 3.6–10.7) and ‘slowly worsening’ (HR 3.4, 95% CI 2.0–5.9) trajectories were associated with an increased risk of KR (p<0.001 for both trajectories) compared to the ‘no change’ trajectory. The difference in KR free survival of the ‘improving’ (HR 0.9, 95% CI 0.3–2.6) trajectory was statistically insignificant from the ‘no change’ trajectory.

### Association of pain medication use with HRQoL trajectory groups

**Fig 4:**
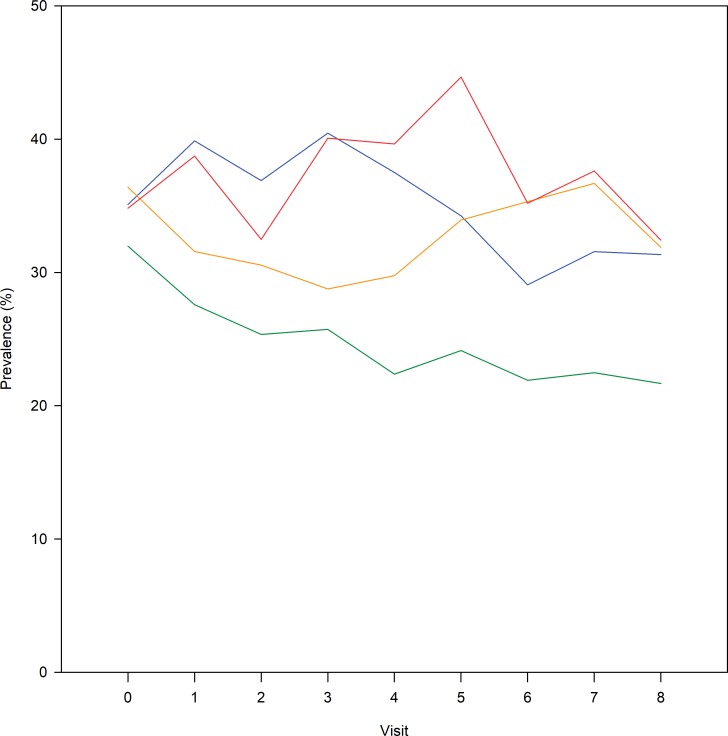
Frequent pain medication users (prevalence, %) by HRQoL trajectory groups. Pooled answers regarding the use of strong pain medications, acetaminophen and nonsteroidal anti-inflammatory drugs. Answers were dichotomized (frequent pain medication user / nonuser of pain medication). HRQoL change (MIC, a minimally important reduction in KOOS QoL) trajectory groups: no change in KOOS QoL (green line); improving KOOS QoL after decline (blue line); slowly worsening KOOS QoL (yellow line); rapidly worsening KOOS QoL (red line).

Fig 4 presents the use of pain medication in the trajectory groups during the follow-up. At baseline, 32% in the ‘no change’ group, 35% in the ‘improving’ group, 36% in the ‘slowly worsening’ group, and 35% of the participants in the ‘rapidly worsening’ trajectories, respectively, were classified as frequent pain medication users. These proportions varied during the follow-up (‘no change’: 22–32%; ‘improving’: 29–40%; ‘slowly worsening’: 29–37%; ‘rapidly worsening’: 32–45%). The frequent use of pain medication in the ‘no change’ trajectory was less common than in the three other trajectories (p<0.001, adjusted for age, gender and baseline medication use).

## Discussion

We identified four distinct HRQoL trajectory groups, which described the probability of experiencing an important reduction in disease-specific HRQoL in subjects with mild knee OA or at increased risk at baseline. The observed patterns during the 8-year follow-up were ‘no change’, ‘improving’ KOOS QoL after an initial decline, ‘slowly worsening’, and ‘rapidly worsening’. We found that female gender, being overweight, smoking, knee pain and lower income at baseline were associated with belonging to the ‘rapidly worsening’ trajectory. Our results also demonstrate that frequent pain medication use and KR are more common among people belonging to the ‘slowly worsening’ or ‘rapidly worsening’ trajectories.

The practical relevance of the present study was to demonstrate that different HRQoL change pathways exist, and that there is a difference between the members assigned into the groups with regard to both risk factors and the need for interventions. In other words, it may be possible to identify persons at risk of suffering a worsening in HRQoL, and who could benefit from early interventions. As our aim was to identify those experiencing an HRQoL worsening in the early stages of knee OA, we included persons with mild knee OA or at increased risk. In chronic conditions such as knee OA, the main aim of intervention is to maintain or improve the QoL of patients [33]. In the future, the HRQoL of patients with knee OA could be combined with a recently developed computational model that is able to predict articular cartilage degeneration during the progression of OA and even differentiate different K-L grade groups from each other based on the patient’s baseline information [34,35]. This would enable simultaneous prediction of OA and expected HRQoL outcomes of comparative interventions from the available baseline information when planning optimal treatment for those patients with OA or at risk of developing the disease (e.g., targeting of treatment to specific patients). Also, this information would enable economic evaluation of different OA treatments.

Our results are consistent with published reports showing that knee OA takes different pathways rather than following a uniform course. Previously, it has been demonstrated that there are different trajectories of pain and functional limitations in knee OA [8–12]. Our results are also in accordance with the previous studies showing that female gender, being overweight, experiencing pain and lower socioeconomic status are associated with poorer outcomes in knee OA [8–12,36]. Female gender and overweight are also well-known risk factors for the onset of knee OA [3] and the experience of pain has been shown to be an important factor in OA-related QoL [15–19].

Older age is also a risk factor of knee OA [3]. However, we found that the oldest age group was not associated with belonging to the ‘rapidly worsening’ trajectory. One possible explanation may be that aged people have adapted to pain and the functional limitations imposed by knee OA. Our results also suggest that smoking is associated with HRQoL worsening. This may be explained by a higher risk of musculoskeletal pain among smokers as compared to non-smokers, although it has been claimed that smoking may modestly protect from the development of radiographic knee and hip OA possibly via lower BMI [37].

Our study confirms previous results that the HRQoL change trajectories are associated with the need for treatment [9,11]. We found that the cumulative incidence of KR during the extended 10-year follow-up was higher among individuals belonging to the ‘rapidly worsening’ (8.2%) trajectory as compared to the ‘no change’ (1.4%) trajectory. The prevalence of frequent pain medication use was 32–45% among persons in the ‘rapidly worsening’ trajectory which was more than the 22–32% in the ‘no change’ trajectory during the 8-year follow-up. Previous studies have reported that the proportion of persons undergoing KR in the worst pain or poorest physical activity trajectories varies from 4% to 20% during 5–6 years of follow-up [9–11]. The prevalence of frequent pain medication use has ranged from 54% to 73% in those subjects in the poorest pain trajectories [9,11]. The comparability between our study and previous studies is, however, complicated due to the different outcomes modeled in the trajectory analyses, different inclusion criteria, and variation in phrasing of pain medication questions, although all studies utilized the OAI dataset [9,11]. In addition, our study covered pain medication use during follow-up while others have reported only baseline pain medication use.

The strength of our study is that we quantified HRQoL trajectories in early knee OA with GBTM, and in conjunction with analyses of KRs and frequent pain medication use. Secondly, our analyses are based on the KOOS QoL measure which encompasses the person’s own experience of HRQoL and daily functioning in relation to both knees. Thirdly, our study applied a long OAI follow-up period of eight years, and information of KRs was captured during a 10-year follow-up period of medical records.

The results of the present study should be interpreted in the light of some limitations. First, the cases worsening at every follow-up time point and the cases worsening at one follow-up time point only and remaining at that level afterwards, were classified similarly. However, comparing the KOOS QoL score with the baseline score at each follow-up time-point enabled long-term predictions, in other words, prediction of the knee OA patient’s status in the future as compared to the baseline. Second, we performed a two-stage latent membership analysis, where the response variable (change in KOOS QoL) was used to categorize the individuals in the first stage, and in the second stage, a multinomial logistic regression was utilized to identify cross-group differences [38]. A joint probability model for response variable patterns and risk factors carries less uncertainty than a two-stage model in assigning the individuals into the appropriate trajectories. However, the joint probability model would have resulted in a more complex mixture model, and the covariates would have shaped the trajectories. The advantage of the two-stage model is that it can be considered as a risk factor analysis and our aim was to identify those factors that explain the latent trajectory membership. Third, as MIC is defined as the smallest difference in instrument scores which the patient can perceive and which mandates a change in patient treatment [39], it is evident that there is no single value for MIC but more likely a range of MICs [40]. We used the value of 10 points as a cut-point for MIC as the smallest change in KOOS QoL score that would be clinically important to the subject. However, estimates for MIC-values vary in the different studies and also according to age, patient group, and treatment. The strength of MIC values is that they simplify the interpretation of whether or not the change in HRQoL score is clinically meaningful; for this reason threshold scores are commonly applied in HRQoL literature. By using a dichotomous MIC-variable, we aggregated the subjects into the same trajectory group regardless of the baseline KOOS QoL score level but with regard to the fact that they were in a similar KOOS QoL pathway. If we had utilized a continuous KOOS QoL score, it would have resulted in aggregating those individuals with similar scores, but with possibly dissimilar pathways. Fourth, the KOOS QoL measure has items of awareness, avoidance, confidence and overall difficulties related to the knee(s), and hence, KOOS QoL may not measure HRQoL, but rather activity and function. On the other hand, HRQoL reflects also the possible deficits in the subject’s physical activity. Fifth, it is uncertain how individuals respond to these questionnaires in annual query reiterations e.g. after they have possibly made an adaptation to pain and accepted the functional decline as a fact of life. In addition, an annual inquiry of KOOS QoL maybe somewhat too infrequent as knee OA symptoms may fluctuate [41]. However, OAI is a comprehensive and reliable follow-up study of patients with knee OA or at an increased risk of developing this disease.

In our study of HRQoL in an early knee OA population, we detected four diverse trajectories with HRQoL worsening being associated with several risk factors (e.g., female gender, overweight, and knee pain). We observed that surgical and pharmacological interventions were more common in those individuals in the poorer trajectories indicating that HRQoL does reflect the need for OA treatment. These findings may have implications for targeting appropriate interventions to specific knee OA patient groups.

## Supporting information

S1 Table Specific OAI datasets applied in the study. (DOCX)

S2 Table BIC-values and estimated group sizes for the tested group-based trajectory models with 2–6 groups. ^a^Bayesian information criterion.
(DOCX)

S3 Table Diagnostic criteria of the final trajectory model. ^a^There should be a close correspondence between the model estimate of group probability and the proportion of individuals classified in the group (classification based on the maximum posterior probability assignment rule). ^b^The average of the posterior probabilities of individuals assigned to a group. This should be at least 0.7. ^c^Should be ≥5.0.
(DOCX)

S4 Table Baseline demographics and clinical characteristics of participants in the study stratified by trajectory groups with different HRQoL changes (MIC, a minimally important reduction in KOOS QoL). Values are *N* (%) unless otherwise noted. ^a^Body Mass Index (kg/m^2^). ^b^Quality of Life subscale of the Knee injury and Osteoarthritis Outcome Score (range 0–100). ^c^Physical Activity Scale for the Elderly (range 0–526). ^d^Center for Epidemiologic Studies Depression Scale (range 0–60).
(DOCX)
