# Supplementary material for: Eight-year trajectories of changes in health-related quality of life in knee osteoarthritis: Data from the Osteoarthritis Initiative (OAI)
Source: PLoS One. 2019 Jul 19;14(7):e0219902. doi: 10.1371/journal.pone.0219902 (PMC6641160; doi:10.1371/journal.pone.0219902)
Supplement: S1 Table — (DOCX) [file pone.0219902.s001.docx]

**S1 Table**

| Variable | Dataset label |
| --- | --- |
| Gender | enrollees |
| Kellgren-Lawrence grade | kXR_SQ_BU00 |
|  | kXR_SQ_BU01 |
|  | kXR_SQ_BU03 |
|  | kXR_SQ_BU05 |
|  | kXR_SQ_BU06 |
|  | kXR_SQ_BU08 |
|  | kXR_SQ_BU10 |
| Knee replacement | outcomes99 |
| Other variables | AllClinical00 |
|  | AllClinical01 |
|  | AllClinical03 |
|  | AllClinical05 |
|  | AllClinical06 |
|  | AllClinical07 |
|  | AllClinical08 |
|  | AllClinical09 |
|  | AllClinical10 |
