# Supplementary material for: Eight-year trajectories of changes in health-related quality of life in knee osteoarthritis: Data from the Osteoarthritis Initiative (OAI)
Source: PLoS One. 2019 Jul 19;14(7):e0219902. doi: 10.1371/journal.pone.0219902 (PMC6641160; doi:10.1371/journal.pone.0219902)
Supplement: S2 Table — aBayesian information criterion. (DOCX) [file pone.0219902.s002.docx]

**S2 Table**

| Number of groups | Polynomial order | BIC^a^ | Estimated group sizes (%) | | | | | |
| --- | --- | --- | --- | --- | --- | --- | --- | --- |
| 2 | 22 | –9773.05 | 73.21 | 26.79 | – | – | – | – |
| 3 | 222 | –9634.02 | 58.46 | 32.08 | 9.46 | – | – | – |
| 4 | 2222 | –9607.74 | 18.43 | 58.89 | 12.94 | 9.75 | – | – |
| 5 | 22222 | –9620.38 | 22.61 | 42.65 | 15.87 | 10.15 | 8.72 | – |
| 6 | 222222 | –9611.66 | 16.53 | 57.89 | 11.00 | 8.23 | 3.60 | 2.74 |
| 2 | 33 | –9778.91 | 73.22 | 26.78 | – | – | – | – |
| 3 | 333 | –9641.57 | 58.53 | 32.06 | 9.41 | – | – | – |
| 4 | 3333 | –9608.98 | 19.45 | 59.45 | 11.58 | 9.51 | – | – |
| 5 | 33333 | –9620.36 | 19.54 | 52.87 | 8.33 | 12.47 | 6.79 | – |
| 6 | 333333 | –9632.02 | 5.30 | 53.48 | 15.92 | 8.15 | 10.76 | 6.40 |
| 4 | 3031 | –9595.20 | 19.11 | 59.54 | 11.85 | 9.50 | – | – |
