# Supplementary material for: Eight-year trajectories of changes in health-related quality of life in knee osteoarthritis: Data from the Osteoarthritis Initiative (OAI)
Source: PLoS One. 2019 Jul 19;14(7):e0219902. doi: 10.1371/journal.pone.0219902 (PMC6641160; doi:10.1371/journal.pone.0219902)
Supplement: S3 Table — aThere should be a close correspondence between the model estimate of group probability and the proportion of individuals classified in the group (classification based on the maximum posterior probability assignment rule). bThe average of the posterior probabilities of individuals assigned to a group. This should be at least 0.7. cShould be ≥5.0. (DOCX) [file pone.0219902.s003.docx]

#### **S3 Table**

| Group | Model estimate of group probability | Proportion classified^a^ | Average pp^b^ | Odds correct classification^c^ |
| --- | --- | --- | --- | --- |
| No change in KOOS QoL | 0.595 | 0.629 | 0.900 | 6.09 |
| Slowly worsening KOOS QoL | 0.191 | 0.171 | 0.718 | 10.78 |
| Improving KOOS QoL after decline | 0.119 | 0.104 | 0.664 | 14.70 |
| Rapidly worsening KOOS QoL | 0.095 | 0.095 | 0.831 | 46.81 |
