# Supplementary material for: Eight-year trajectories of changes in health-related quality of life in knee osteoarthritis: Data from the Osteoarthritis Initiative (OAI)
Source: PLoS One. 2019 Jul 19;14(7):e0219902. doi: 10.1371/journal.pone.0219902 (PMC6641160; doi:10.1371/journal.pone.0219902)
Supplement: S4 Table — Values are N (%) unless otherwise noted. aBody Mass Index (kg/m2). bQuality of Life subscale of the Knee injury and Osteoarthritis Outcome Score (range 0–100). cPhysical Activity Scale for the Elderly (range 0–526). dCenter for Epidemiologic Studies Depression Scale (range 0–60). (DOCX) [file pone.0219902.s004.docx]

**S4 Table**

| Variable | Category | No change in KOOS QoL | Improving KOOS QoL after decline | Slowly worsening KOOS QoL | Rapidly worsening KOOS QoL | Total |
| --- | --- | --- | --- | --- | --- | --- |
| Participants |  | 1921 (62.9) | 319 (10.4) | 522 (17.1) | 291 (9.5) | 3053 (100.0) |
| Cohort | Incidence | 1584 (82.5) | 268 (84.0) | 431 (82.6) | 231 (79.4) | 2514 (82.3) |
|  | Progression | 337 (17.5) | 51 (16.0) | 91 (17.4) | 60 (20.6) | 539 (17.7) |
| Age (years) | 45–54 | 647 (33.7) | 106 (33.2) | 160 (30.7) | 95 (32.6) | 1008 (33.0) |
|  | 55–64 | 633 (33.0) | 103 (32.3) | 169 (32.4) | 113 (38.8) | 1018 (33.3) |
|  | 65–79 | 641 (33.4) | 110 (34.5) | 193 (37.0) | 83 (28.5) | 1027 (33.6) |
|  | Total | 1921 (100.0) | 319 (100.0) | 522 (100.0) | 291 (100.0) | 3053 (100.0) |
|  | Mean (SD) | 60.1 (9.0) | 60.5 (9.0) | 61.0 (9.2) | 59.7 (8.6) | 60.2 (9.0) |
| Gender | Female | 1139 (59.3) | 207 (64.9) | 313 (60.0) | 190 (65.3) | 1849 (60.6) |
|  | Male | 782 (40.7) | 112 (35.1) | 209 (40.0) | 101 (34.7) | 1204 (39.4) |
|  | Total | 1921 (100.0) | 319 (100.0) | 522 (100.0) | 291 (100.0) | 3053 (100.0) |
| BMI^a^ | <25 | 561 (29.2) | 72 (22.6) | 139 (26.6) | 52 (17.9) | 824 (27.0) |
|  | 25 to <30 | 766 (39.9) | 126 (39.5) | 201 (38.5) | 118 (40.5) | 1211 (39.7) |
|  | 30 to <35 | 442 (23.0) | 88 (27.6) | 139 (26.6) | 93 (32.0) | 762 (25.0) |
|  | ≥35 | 150 (7.8) | 33 (10.3) | 43 (8.2) | 28 (9.6) | 254 (8.3) |
|  | Total | 1919 (100.0) | 319 (100.0) | 522 (100.0) | 291 (100.0) | 3051 (100.0) |
|  | Mean (SD) | 27.9 (4.7) | 28.6 (4.8) | 28.3 (4.5) | 29.1 (4.6) | 28.1 (4.7) |
|  | Missing | 2 | 0 | 0 | 0 | 2 |
| Race | White | 1554 (81.0) | 256 (80.3) | 434 (83.1) | 237 (81.4) | 2481 (81.3) |
|  | Non-White | 364 (19.0) | 63 (19.7) | 88 (16.9) | 54 (18.6) | 569 (18.7) |
|  | Total | 1918 (100.0) | 319 (100.0) | 522 (100.0) | 291 (100.0) | 3050 (100.0) |
|  | Missing | 3 | 0 | 0 | 0 | 3 |
| Socioeconomic status | | | | | | |
| Education | None/Primary | 688 (36.0) | 120 (38.0) | 198 (38.2) | 129 (44.5) | 1135 (37.4) |
|  | Secondary | 598 (31.3) | 95 (30.1) | 161 (31.0) | 82 (28.3) | 936 (30.8) |
|  | Tertiary | 625 (32.7) | 101 (32.0) | 160 (30.8) | 79 (27.2) | 965 (31.8) |
|  | Total | 1911 (100.0) | 316 (100.0) | 519 (100.0) | 290 (100.0) | 3036 (100.0) |
|  | Missing | 10 | 3 | 3 | 1 | 17 |
| Income | <$25k | 197 (10.9) | 41 (13.8) | 57 (11.9) | 48 (17.5) | 343 (12.0) |
|  | $25k to <$50k | 442 (24.6) | 64 (21.5) | 121 (25.3) | 73 (26.6) | 700 (24.6) |
|  | $50k to <$100k | 673 (37.4) | 115 (38.7) | 178 (37.2) | 101 (36.9) | 1067 (37.5) |
|  | $100k or greater | 488 (27.1) | 77 (25.9) | 122 (25.5) | 52 (19.0) | 739 (25.9) |
|  | Total | 1800 (100.0) | 297 (100.0) | 478 (100.0) | 274 (100.0) | 2849 (100.0) |
|  | Missing | 121 | 22 | 44 | 17 | 204 |
| Living status | Living with someone else | 1501 (78.6) | 245 (77.5) | 407 (78.4) | 220 (76.1) | 2373 (78.2) |
|  | Living alone | 408 (21.4) | 71 (22.5) | 112 (21.6) | 69 (23.9) | 660 (21.8) |
|  | Total | 1909 (100.0) | 316 (100.0) | 519 (100.0) | 289 (100.0) | 3033 (100.0) |
|  | Missing | 12 | 3 | 3 | 2 | 20 |
| Clinical characteristics of knee | | | | | | |
| Knee pain severity | No pain (0–1) | 559 (29.2) | 113 (35.4) | 209 (40.0) | 120 (41.2) | 1001 (32.9) |
|  | Mild pain (2–3) | 613 (32.0) | 87 (27.3) | 149 (28.5) | 89 (30.6) | 938 (30.8) |
|  | Moderate pain (4–10) | 743 (38.8) | 119 (37.3) | 164 (31.4) | 82 (28.2) | 1108 (36.4) |
|  | Total | 1915 (100.0) | 319 (100.0) | 522 (100.0) | 291 (100.0) | 3047 (100.0) |
|  | Mean (SD) | 3.1 (2.5) | 3.0 (2.8) | 2.7 (2.6) | 2.5 (2.5) | 3.0 (2.6) |
|  | Missing | 6 | 0 | 0 | 0 | 6 |
| Knee injuries | No | 1117 (58.9) | 189 (60.4) | 321 (62.6) | 186 (64.8) | 1813 (60.2) |
|  | Yes | 781 (41.1) | 124 (39.6) | 192 (37.4) | 101 (35.2) | 1198 (39.8) |
|  | Total | 1898 (100.0) | 313 (100.0) | 513 (100.0) | 287 (100.0) | 3011 (100.0) |
|  | Missing | 23 | 6 | 9 | 4 | 42 |
| Knee surgical history | No | 1642 (85.7) | 268 (84.0) | 441 (84.6) | 251 (86.6) | 2602 (85.4) |
|  | Yes | 274 (14.3) | 51 (16.0) | 80 (15.4) | 39 (13.4) | 444 (14.6) |
|  | Total | 1916 (100.0) | 319 (100.0) | 521 (100.0) | 290 (100.0) | 3046 (100.0) |
|  | Missing | 5 | 0 | 1 | 1 | 7 |
| KOOS QoL^b^ | Mean (SD) | 67.6 (20.0) | 74.9 (20.1) | 75.6 (18.9) | 80.9 (17.2) | 71.0 (20.1) |
| Other factors | | | | | | |
| Charlson comorbidity index | 0 | 1484 (78.0) | 227 (73.0) | 408 (78.9) | 218 (76.2) | 2337 (77.5) |
|  | ≥1 | 419 (22.0) | 84 (27.0) | 109 (21.1) | 68 (23.8) | 680 (22.5) |
|  | Total | 1903 (100.0) | 311 (100.0) | 517 (100.0) | 286 (100.0) | 3017 (100.0) |
|  | Mean (SD) | 0.3 (0.8) | 0.4 (0.9) | 0.3 (0.7) | 0.4 (1.0) | 0.4 (0.8) |
|  | Missing | 18 | 8 | 5 | 5 | 36 |
| PASE^c^ (quintiles) | 0–90 | 396 (20.7) | 66 (20.9) | 81 (15.5) | 66 (22.8) | 609 (20.1) |
|  | 91–134 | 368 (19.3) | 69 (21.8) | 114 (21.9) | 53 (18.3) | 604 (19.9) |
|  | 135–175 | 364 (19.1) | 63 (19.9) | 121 (23.2) | 60 (20.8) | 608 (20.0) |
|  | 176–237 | 383 (20.1) | 59 (18.7) | 100 (19.2) | 66 (22.8) | 608 (20.0) |
|  | 238–526 | 399 (20.9) | 59 (18.7) | 105 (20.2) | 44 (15.2) | 607 (20.0) |
|  | Total | 1910 (100.0) | 316 (100.0) | 521 (100.0) | 289 (100.0) | 3036 (100.0) |
|  | Mean (SD) | 166.2 (84.6) | 162.9 (83.9) | 166.5 (77.2) | 158 (80.2) | 165.1 (82.9) |
|  | Missing | 11 | 3 | 1 | 2 | 17 |
| CES-D^d^ | <16 | 1738 (91.4) | 279 (88.9) | 467 (90.5) | 260 (89.7) | 2744 (90.8) |
|  | ≥16 | 163 (8.6) | 35 (11.1) | 49 (9.5) | 30 (10.3) | 277 (9.2) |
|  | Total | 1901 (100.0) | 314 (100.0) | 516 (100.0) | 290 (100.0) | 3021 (100.0) |
|  | Mean (SD) | 6.2 (6.7) | 7.2 (7.3) | 6.5 (6.4) | 7.0 (7.7) | 6.4 (6.8) |
|  | Missing | 20 | 5 | 6 | 1 | 32 |
| Smoking | Never | 899 (47.4) | 141 (45.2) | 227 (44.1) | 118 (41.1) | 1385 (46.0) |
|  | Former | 826 (43.6) | 144 (46.2) | 233 (45.2) | 130 (45.3) | 1333 (44.3) |
|  | Current | 170 (9.0) | 27 (8.7) | 55 (10.7) | 39 (13.6) | 291 (9.7) |
|  | Total | 1895 (100.0) | 312 (100.0) | 515 (100.0) | 287 (100.0) | 3009 (100.0) |
|  | Missing | 26 | 7 | 7 | 4 | 44 |
